# On authentication of cell lines

**Published:** 2013-08-27

**Authors:** The Editors of Molecular Vision

## The RGC-5 cell line may have never existed

The RGC-5 cell line was first described in 2001 as being an immortalized cell line derived from rat retinal ganglion cells (RGCs) [1]. At least 230 articles have been published in which this cell line was used to test hypotheses. Eighteen of these were published in *Molecular Vision* [2-19].

Work recently published by several of the co-authors of the original RGC-5 paper indicates that the cell line may have never existed outside of the originating laboratory [20]. Cryopreserved RGC-5 passages, some as early as the second passage (P2), were obtained from the originating laboratory. These cells were found to be genetically identical to 661W cells, an immortalized mouse photoreceptor cell line [21] that was in use in the originating laboratory at the time that the RGC-5 cell line was being developed [20]. It is likely that from P2 on, all RGC-5 clones were instead 661W cells [20]. We commend the authors and *Investigative Ophthalmology and Vision Science* for providing this correction.

Previous concerns about the identity of the RGC-5 cell line, some published [22,23], resulted in a policy of *Molecular Vision*, which was until now:

"Cells of questionable characterization cannot be used to test hypotheses specifically concerning retinal ganglion cells. Manuscripts reporting experiments in which putative RGC-5 cells are used must include data that demonstrate that the RGC-5 cells, as used under the culture conditions of the other experiments reported in the manuscript, are of rat origin and express genes and proteins that are specific to retinal ganglion cells (i.e., they should be positive for Thy-1, Brn-3C, Neuritin, NMDA receptor, GABA-B receptor, and synaptophysin expression and negative for GFAP, HPC-1, and 8A1, as reported in Krishnamoorthy et al.)" - from Molecular Vision Instructions to Authors, dated January 1, 2013 to August 23, 2013).

This policy has been revised to: "*New manuscripts containing data derived from RGC-5 cells will be editorially rejected without review.*"

## *Molecular Vision* policy on use of cell lines: Compulsory authentication

The editors of *Molecular Vision* recognize that the proper use of immortalized cell lines can result in the rapid generation of substantial data in testing many hypotheses. However, due to continual problems of misidentification of cell lines for at least 55 years [24-28], including estimates of mammalian cell misidentification of 15%-35% [27,29,30], the new *Molecular Vision* policy for reporting data based on immortalized cell lines is:

"Manuscripts reporting experiments in which immortalized cell lines are used must include data, documentation, or citations that demonstrate that the actual cells used in the experiments reported in the manuscript exhibit the correct phenotype and genotype. It must be demonstrated that the cells actually used are of the correct species of origin, the correct sex and genotype, and express genes and gene products that are specific to the pertinent cell type. Where possible, phenotype analysis should include the effects of differentiation. Cells used in experiments should be within a few passages of authentication (typically five passages). These standards hold even if the cell lines are considered "established" and were obtained from reputable sources. A statement of cell handling protocol that includes passage information and authentication data, certification documentation, and/or citation of published authentication by the co-authors must be provided in Methods sections of submitted manuscripts. These will be part of the freely-available article." - Molecular Vision Instructions to Authors, August 27, 2013.

## Meeting these criteria

Authors are encouraged to study the history and consequences of misidentification of cell lines and the array of solutions available to avoid this chronic problem. Several excellent reviews and primary papers are available (e.g., [22,24-28,31]). Even a cursory reading of these sources provides insight into the historical lack of scientific rigor and expensive outcomes in terms of research monies and careers that has plagued the misuse of immortalized cell lines.

The editors of *Molecular Vision* agree with The International Cell Line Authentication Committee (ICLAC) and the National Institutes of Health (NIH) in their statements of the need for cell line authentication (NOT-OD-08-017) and approaches to accomplish this authentication [25]. The ICLAC provides guidelines for incorporating authentication into good tissue-culture practice (Advice to Scientists: Incorporating Authentication into Everyday Culture Practice). A similar online resource is published by the National Center for Biotechnology Information [30]. Briefly, once a cell line is authenticated, it is expanded to create a "master stock" of cell aliquots for cryopreservation. An aliquot from the master stock is expanded to create a "distribution stock" of cell aliquots. An aliquot from the distribution stock is expanded into aliquots of cells that are used in the actual experiments reported in submitted manuscripts. Thus, by best practices, the cells actually used in experiments should be within five passages of authentication (this is not five passages from the initial establishment of the cell line, which may have occurred many passages earlier). These guidelines must be followed and documented in order for data based on the use of immortalized cell lines to be published in *Molecular Vision*. Exemptions for incidental use of immortalized cells (e.g., simple expression confirmation assays) might occur following scrutiny by reviewers and editors. The journal’s Instructions to Authors will contain the above policy statement and supporting details, including examples of potential exceptions.

Authentication itself principally involves short-tandem-repeat (STR) profiling using standards and protocols developed by the American National Standards Institute for human cell lines [30,32] and by the National Institute of Standards and Technology for mouse cell lines [33]. The articles reporting the re-characterization of the RGC-5 cell line also provide insight into authentication approaches [20,22,23]. Cell lines also may be authenticated by replicating the experiments published for initial characterization. Authors can either provide data in their submitted manuscripts demonstrating that cell lines were authenticated by these various standards, or they can contract with external services (e.g., ATCC, Promega, Identicell, DSMZ, Genetica, and others not listed) to provide authentication. If contract services are used, documentation from the service must be supplied to *Molecular Vision* as part of the manuscript submission. Note that only some, but not all, cell lines sold by commercial suppliers are authenticated. It is the authors' responsibility to confirm authentication.

## Vision researchers should lead in ending misidentification

The need for authentication of immortalized cell lines has previously been called for in our field [34]. However, the use of misidentified cell lines has harmed the vision research community. Resources were wasted and misinformation was propagated (which itself will require further expenditures for remediation). *Molecular Vision* has committed to halt these problems by enforcing the above-stated policy. However, a wider approach is needed to effect change. To that end, we have worked with the editors of *Experimental Eye Research*, the flagship journal of the International Society for Eye Research (ISER) and *Investigative Ophthalmology and Visual Science*, the flagship journal of the Association for Research in Vision and Ophthalmology (ARVO), in formulating our policy. The policies of these journals are presented in recent editorials and in the journals' Instructions to Authors. We encourage other vision research journals to consider joining in this editorial effort to preempt the replication of this type of error in future work by mandating cell line authentication for all submitted manuscripts. Further, we urge scientific and professional organizations to set standards for cell line maintenance and authentication and to develop educational resources (online and at major meetings) to aid their members in meeting these standards. We urge universities to hold similar courses. Finally, we encourage funding agencies to require that cell line maintenance and authentication protocols be incorporated into project proposals.

## Editors-in-Chief

### Molecular Vision

Jeffrey H. Boatright, PhD, FARVOPatrick R. Cammarata, PhD, FARVOJohn M. Nickerson, PhD, FARVOQingjiong Zhang, MD, PhD
